# Long-term effects of gastric bypass and duodenal switch on systemic exposure of atorvastatin

**DOI:** 10.1007/s00464-012-2716-3

**Published:** 2012-12-18

**Authors:** Gunn Signe Jakobsen, Ine Blankenberg Skottheim, Rune Sandbu, Hege Christensen, Jo Røislien, Anders Åsberg, Jøran Hjelmesæth

**Affiliations:** 1 Morbid Obesity Centre, Vestfold Hospital Trust, P.O. Box 2168, 3103 Tønsberg, Norway; 2 School of Pharmacy, University of Oslo, Oslo, Norway; 3 Department of Biostatistics, University of Oslo, Oslo, Norway

**Keywords:** Atorvastatin, Biliopancreatic diversion with duodenal switch, Gastric bypass, Systemic exposure

## Abstract

**Background:**

A previous study of 22 patients undergoing either gastric bypass or duodenal switch showed increased systemic exposure of atorvastatin acid 3–8 weeks after surgery in the majority of patients. This study aimed to investigate the long-term effects on systemic exposure of atorvastatin acid in the same group of patients.

**Methods:**

An 8-h pharmacokinetic investigation was performed a median of 27 months (range 21–45 months) after surgery. Systemic exposure was measured as the area under the plasma concentration versus the time curve from 0 to 8 h postdose (AUC_0–8_). Linear mixed models with AUC_0–8_ as the dependent variable were implemented to assess the effect of time, surgical procedure, and body mass index (BMI) as explanatory variables.

**Results:**

The study enrolled 20 patients. The systemic exposure of atorvastatin acid changed significantly over time (*p* = 0.001), albeit there was substantial variation between subjects. The effect of time was attenuated but remained significant after adjustment for surgical procedure and BMI (*p* = 0.048). The initial AUC_0–8_ increase seen in the majority of patients 3–8 weeks after surgery was normalized long term, with 7 of the 12 gastric bypass patients and 6 of the 8 duodenal switch patients showing decreased AUC_0–8_ compared with preoperative values.

**Conclusions:**

The systemic exposure of atorvastatin showed a significant change over time after bariatric surgery, albeit with large inter- and intraindividual variations. The findings indicate that patients using atorvastatin or drugs with similar pharmacokinetic properties should be monitored closely for both therapeutic effects and adverse events the first years after gastric bypass and duodenal switch. ClinicalTrial NCT00331565.

Bariatric surgery is widely used to treat morbid obesity given that it provides sustained weight loss and leads to improvement in both quality of life and obesity-related comorbidities [14, 27]. Gastric bypass and biliopancreatic diversion with duodenal switch (duodenal switch) are two commonly performed procedures [2]. Both procedures bypass the duodenum and proximal small intestine, but the bypass of the small intestine is greater with duodenal switch than with gastric bypass.

The bypass of the proximal small intestine affects the absorption of several ingested nutrients and vitamins [1]. The bioavailability of orally administered drugs also can be affected [7, 18].

We recently have shown that the systemic exposure of atorvastatin increased in the majority of patients the first weeks after both gastric bypass [29] and duodenal switch [28]. The exact mechanism for this is unknown, but we hypothesize that highly active restrictive processes in the small intestine (with regard to drug bioavailability) are bypassed, such as metabolizing enzymes in the intestinal wall. In particular, the CYP450 isoenzymes CYP3A4 and CYP3A5 are involved in restricting the bioavailability of atorvastatin and many other substrate drugs [21]. These CYP enzymes are highly expressed in the proximal intestine and decrease along the small intestine [20].

Patients normally lose weight quickly after bariatric surgery. This process often continues for the first 2 years postoperatively, after which the weight either stabilizes or takes the shape of a small long-term weight gain. No conclusive explanations exist to elucidate why the weight loss stops, but it might partly be explained by adaptive changes in the intestine, changed habits of the patients, or metabolic adaptation [10]. Similar mechanisms also may affect drug bioavailability.

In this study, we aimed to explore possible long-term adaptive changes in the systemic exposure of atorvastatin in patients previously treated with either gastric bypass or duodenal switch [28, 29].

## Materials and methods

We have previously presented the short-term results of this prospective, open, controlled, nonrandomized, single-center study that investigated 22 patients listed for either gastric bypass (*n* = 12) or duodenal switch (*n* = 10) [28, 29]. Pharmacokinetic investigations of atorvastatin acid were performed the day before bariatric surgery (baseline) and repeated in the early postoperative phase (3–8 weeks) (short-term follow-up evaluation).

In the current study, a third pharmacokinetic investigation was performed to assess the long-term effects of the two bariatric procedures on systemic exposure of atorvastatin acid. All patients giving specific informed consent for this long-term follow-up investigation were included in the study.

The inclusion and exclusion criteria have been presented previously [28, 29]. Briefly, adult morbidly obese patients [body mass index (BMI) ≥40 kg/m^2^ or BMI ≥35 kg/m^2^ combined with comorbidity] assigned to undergo either gastric bypass or duodenal switch were included in the study. Additional surgery that could potentially interfere with drug bioavailability was an exclusion criterion for the long-term follow-up study. Patients treated with substances that could affect the pharmacokinetics of atorvastatin (listed in previous publications [28, 29]) were not eligible for inclusion in the study.

All the patients were treated with statins on clinical indication before surgery. Patients who used other types of statins were switched to atorvastatin (Lipitor). At both baseline and short- and long-term follow-up evaluations, all the patients received the same dose of atorvastatin for at least 14 days to be in steady-state condition at the time of the investigations. In addition, they completed a drug diary. If a patient had withdrawn from statin treatment or changed the dose since the last investigation, he or she was reintroduced to the same dose of atorvastatin given each morning 2 weeks before the investigation.

The study was conducted in accordance with international and national laws and guidelines. Approvals were obtained from the regional ethics committee and all relevant Norwegian authorities. The study is registered at ClinicalTrial NCT00331565.

### Outcome

The primary outcome was the change in systemic exposure of atorvastatin acid, assessed by the area under the plasma concentration versus the time curve from 0 to 8 h postdose (AUC_0–8_) from baseline (before surgery) via short-term (3–8 weeks) to long-term (21–45 months) follow-up evaluation. Changes in maximal concentration (*C*_max_) and time to reach* C*_max_ (t_max_) for atorvastatin acid also were assessed. The main explanatory variables were time after surgery, surgical procedure, and BMI.

### Surgical procedures

**Fig. 1:**
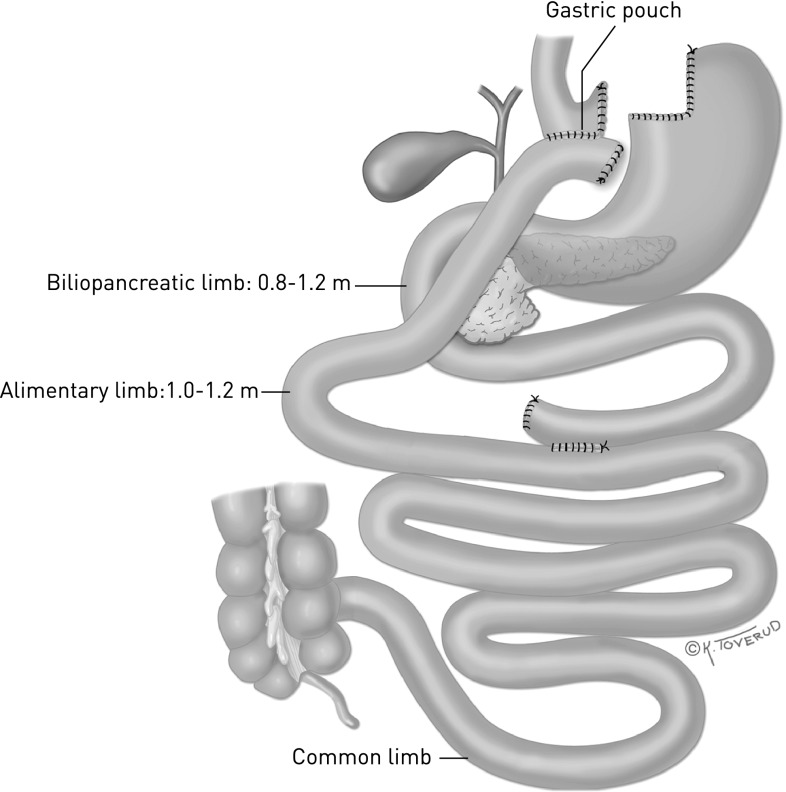
Gastric bypass. By Kari C. Toverud (reproduced from reference [13])

The surgical procedure used was either laparoscopic gastric bypass or biliopancreatic diversion with duodenal switch. Detailed descriptions of the procedures have been presented elsewhere [13, 28, 29]. In short, the gastric bypass was performed by making a small gastric pouch, an 80 cm biliopancreatic limb, and a 120 cm alimentary limb. The remaining part (not measured) of the small intestine is the common channel in which food is mixed with gastric, bile, and pancreatic juices (Fig. 1).

**Fig. 2:**
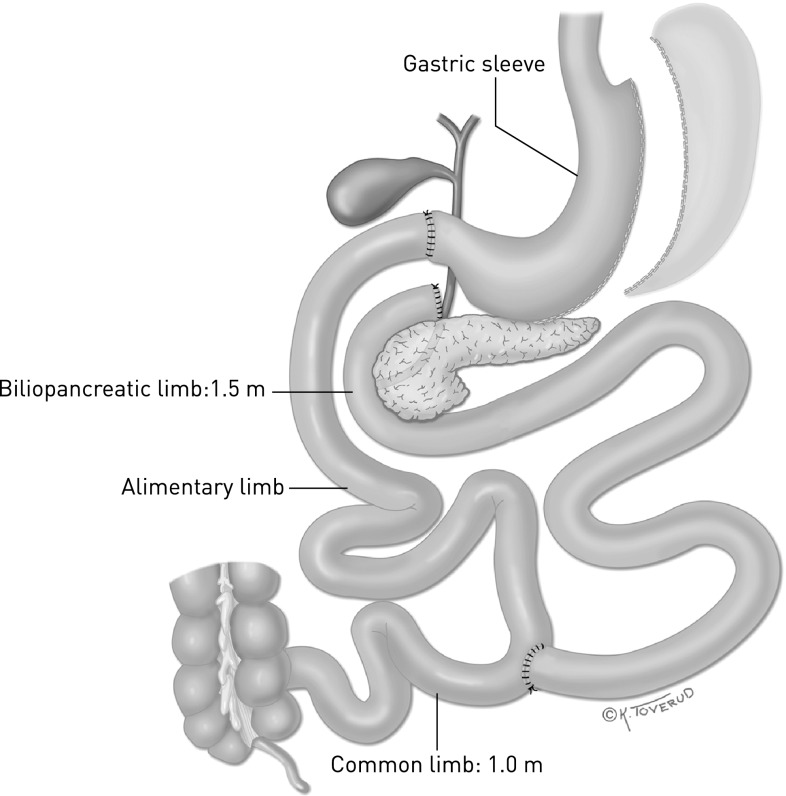
Biliopancreatic diversion with duodenal switch. By Kari C. Toverud (reproduced from reference [13])

The duodenal switch included a vertical sleeve gastrectomy preserving a normal pyloric function. The jejunum was fully bypassed, and an anastomosis was made between the duodenum (proximal to the papilla Vateri) and the small intestine 2.5 m from the ileocecal valve. The entero-enteroanastomosis was made 1 m from the ileocoecal valve so that food was exposed to bile and pancreatic juice only in the last 100 cm of the ileum, the common channel (Fig. 2).

### Pharmacokinetics and bioanalysis

A 24-h pharmacokinetic investigation was performed the day before bariatric surgery (baseline), followed by an 8 h pharmacokinetic investigation short term (3–8 weeks) after surgery as previously reported [28, 29]. In this study, an additional 8-h pharmacokinetic investigation was conducted long term (>21 months) after surgery. As a part of this pharmacokinetic investigation, patients reported to the hospital after an overnight fast (water was permitted but no food, drugs, nicotine, or caffeine). A standard hospital breakfast was served 2-h after atorvastatin administration, and there were no food restrictions thereafter. Concomitant drugs ordinarily administered in the morning were given at breakfast.

Blood samples for the determination of plasma atorvastatin concentrations were drawn in prechilled heparin vacutainers, kept on ice, and centrifuged at 1,800*g* at 4 °C for 10 min, after which plasma was decanted and stored at −80 °C within 1 h of sampling. Blood samples were drawn at predose, then 0.5, 1, 1.5, 2, 3, 5, 6, and 8 h after atorvastatin administration for all investigations.

Atorvastatin acid was analyzed with a validated high-performance liquid chromatography method using tandem mass spectrometry detection [12, 29]. Briefly, sample preparation was performed by solid-phase extraction on C_18_ cartridges followed by chromatographic separation on a C_18_ analytical column combined with electrospray tandem mass spectrometric detection.

Noncompartmental analyses of pharmacokinetic variables were performed using the actual measured values for* C*_max_ and* t*_max_. The AUC_0–8_ was calculated using the trapezoidal method in Microsoft Office Excel 2007 (Microsoft, Redmond, WA, USA) [29].

### Statistical analyses

Data are presented as mean ± standard deviation unless stated otherwise. The dependent variables all were skewly distributed and log-transformed before statistical analysis. To assess the association of time, surgical procedure, and BMI with the changes in systemic exposure of atorvastatin acid, we fitted two linear mixed models with AUC as the dependent variable: a crude model with time as the only explanatory variable and an adjusted model with time, surgical procedure, BMI, and the interaction between time and surgical procedure as explanatory variables. All *p* values lower than 0.05 were considered statistically significant. Statistical analyses were performed using SPSS version 16.0 (SPSS, Inc., Chicago, IL, USA) and R 2.12 [23].

## Results

All 12 patients (six women) in the gastric bypass group were examined three times: at baseline (the day before surgery) and at short-term (3–8 weeks after surgery) and long-term (21–39 months after surgery) follow-up evaluation. All 10 patients (five women) in the duodenal switch group were examined two times; at baseline and at short-term follow-up evaluation.

After the exclusion of one patient due to reoperation that altered the lengths of the intestinal limbs and one patient who declined to participate, eight duodenal switch patients (4 women) were included in the long-term follow-up study. The median follow-up time was 25 months (range 23–45 months) after the duodenal switch and 27 months (range, 21–45 months) for all the patients. All the patients were Caucasians. Only 8 of the 20 patients were continuing to receive statin treatment at the time of inclusion for the long-term follow-up measurement, with four patients using atorvastatin and four patients using simvastatin. The patient characteristics at baseline and at the last follow-up assessment are shown in Table 1.

**Table 1:** Patient demographics, body mass index (BMI), and comorbidities

	Gastric bypass		Duodenal switch	
	Baseline (*n* = 12)	Long-term follow-up (*n* = 12)	Baseline (*n* = 10)	Long-term follow-up (*n* = 8)
Women	6	6	5	4
Age (years)	52.1 ± 8.6	54.3 ± 8.5	43.6 ± 7.2	45.0 ± 7.3
BMI (kg/m^2^)	44.2 ± 3.8	28.6 ± 4.2	49.4 ± 3.1	28.5 ± 4.3
Statin treatment	12	7	10	1
Type 2 diabetes	9	4	6	0
OSA	6	2	8	0
Hypertension	9	6	6	2

Data are given as mean ± standard deviation or as the number of patients

*OSA* obstructive sleep apnea

**Fig. 3:**
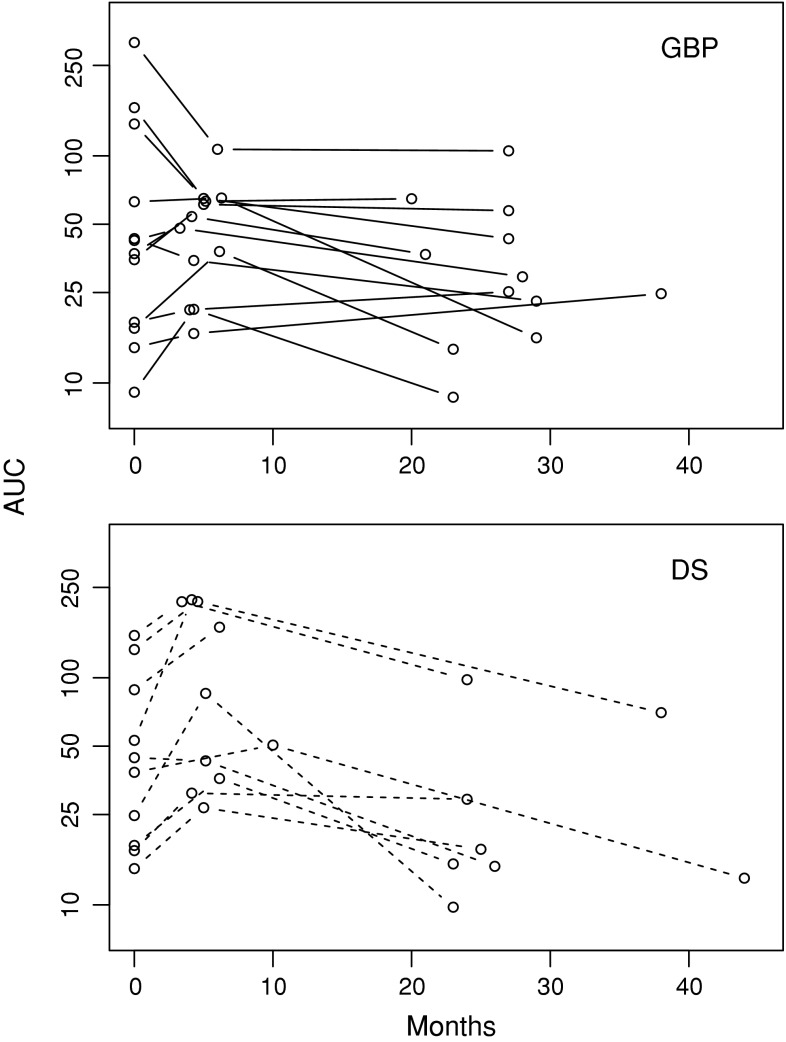
Individual atorvastatin acid AUC_0–8_ (ng h/mL) preoperatively and at both short- and long-term follow-up evaluation. AUC_0–8_, area under the plasma concentration versus the time curve from 0 to 8 h postdose; GBP, gastric bypass; DS, duodenal switch

The systemic exposure of atorvastatin acid varied substantially between the patients (Fig. 3; Table 4) and changed significantly over time (Table 2, crude model; *p* = 0.001). The effect of time was attenuated but remained significant after adjustment for surgical procedure and BMI (Table 2). Furthermore, the surgical procedure times time interaction term was nonsignificant (*p* = 0.221).

**Table 2:** Systemic exposure (AUC_0–8_) of atorvastatin acid, effect of time, surgical procedure, and body mass index (BMI)

	Crude model		Adjusted model	
Explanatory variable	Estimate (95 % CI)	*p* value	Estimate (95 % CI)	*p* value
Time	−0.018 (−0.028 to −0.008)	0.001	−0.024 (−0.046 to −0.001)	0.048
Surgical procedure			0.418 (−0.289 to 1.125)	0.260
Surgical procedure × time			−0.013 (−0.034 to 0.007)	0.221
BMI			−0.021 (−0.055 to 0.013)	0.239

Linear mixed model analysis

*CI* confidence interval, *AUC*_*0–8*_ area under the plasma concentration versus the time curve from 0 to 8 h postdose

Although the systemic exposure of atorvastatin acid varied substantially, 7 of the 12 gastric bypass patients and 6 of the 8 duodenal switch patients showed a decreased AUC_0–8_ long term compared with before surgery (Table 4). The mean long-term/baseline ratio of atorvastatin acid AUC_0–8_ was 0.8 ± 0.5 compared with the short-term/baseline ratio of 1.6 ± 0.9 (Table 3).

**Table 3:** Pharmacokinetic parameters of atorvastatin acid

	Baseline	Ratio short term/baseline	Ratio long term/baseline
AUC (ng*h/mL)	67.4 (73.4)	1.6 (0.9)	0.8 (0.5)
*C*_max_ (ng/mL)	24.5 (37.2)	1.7 (1.4)	0.9 (0.6)
*T*_max_ (h)	1.4 (1.2)	2.2 (1.8)	2.9 (3.1)

Data are given as mean ± standard deviation. Ratios are calculated with short- or long-term values (numerators) and with values at baseline (before surgery) as denominators

*AUC* area under the plasma concentration versus the time curve, *C*_*max*_ maximum plasma concentration, *T*_*max*_ time to reach* C*_max_

The *C*_max_ values for atorvastatin acid showed the same trend as the AUC_0–8_ values in the long term (mixed model: *p* = 0.012; estimate, −0.017; 95 % confidence interval −0.03 to −0.00). On the other hand,* T*_max_ increased with time, from 1.4 ± 1.2 to 2.8 ± 1.9 h (mixed model: *p* = 0.002; estimate, 0.02; 95 % confidence interval −0.01 to −0.04) (Tables 3, 4).

**Table 4:** Atorvastatin acid pharmacokinetic parameters

	AUC_0–8_ (ng*h/mL)			*C*_max_ (ng/mL)			T_max_ (h)		
Patient	Baseline	Ratio short term/baseline	Ratio long term/baseline	Baseline	Ratio short term/baseline	Ratio long term/baseline	Baseline	Ratio short term/baseline	Ratio long term/baseline
Gastric bypass									
9	9	2.3	1.0	2.3	2.0	0.8	1.1	2.7	2.6
10	14	1.2	1.7	5.3	0.7	1.8	0.6	0.8	4.9
8	17	2.2	0.8	4.0	2.2	1.0	2.7	0.2	1.1
7	19	1.1	1.4	5.0	0.7	1.3	0.7	1.7	4.5
5	35	1.8	1.6	5.7	3.0	1.8	5.9	0.3	1.0
12	37	1.5	1.0	8.7	1.3	0.8	1.1	2.7	2.7
3	42	1.1	0.7	7.6	1.6	1.5	0.8	3.8	3.8
2	43	0.8	0.5	7.1	1.0	0.8	1.0	0.8	0.6
1	63	1.0	0.3	10.5	1.5	0.3	1.3	2.5	3.9
6	138	0.5	0.3	33.0	0.6	0.4	1.0	0.5	2.4
11	163	0.4	0.4	117.4	0.2	0.2	1.0	1.5	2.8
4	315	0.3	0.3	133.7	0.2	0.4	1.6	1.9	0.7
Mean ± SD	74 ± 90	1.2 ± 0.7	0.8 ± 0.5	28 ± 46	1.3 ± 0.8	0.9 ± 0.6	1.6 ± 1.5	1.6 ± 1.1	2.6 ± 1.5
Duodenal switch									
3	14	1.9	1.2	3.9	1.5	1.2	0.9	3.4	1.1
4	17	1.8	1.7	2.8	2.3	2.1	2.5	0.8	0.3
8	18	2.0	0.8	3.1	2.6	1.5	1.8	0.3	0.5
10	25	3.4	0.4	6.3	6.2	0.4	0.6	5.5	1.8
5	38	1.3	0.3	7.8	1.8	0.3	0.6	5.6	14.5
1	44	1.0	0.3	15.9	0.5	0.3	0.6	3.6	2.5
2	53	4.2	ND	13.5	4.2	ND	0.5	6.0	ND
6	89	1.9	ND	24.2	1.6	ND	2.4	1.3	ND
9	133	1.6	0.5	42.6	1.0	0.4	0.5	1.3	4.1
7	153	1.4	0.6	78.5	0.8	0.2	1.4	2.2	3.5
Mean ± SD	59 ± 59	2.0 ± 1.0	0.7 ± 0.5	20 ± 24	2.2 ± 1.7	0.8 ± 0.7	1.2 ± 0.8	3.0 ± 2.1	3.5 ± 4.7

*AUC*_*0–8*_ area under the plasma concentration versus the time curve from 0 to 8 h postdose, *C*_*max*_ maximum plasma concentration, *T*_*max*_ time to reach C_max_, *SD* standard deviation, *ND* Not Done

## Discussion

The current prospective study of patients undergoing bariatric surgery shows a significant change in atorvastatin systemic exposure over time. Although large interindividual variations may limit the generalizability of the study, the long-term results show that the initial increase in atorvastatin systemic exposure observed in the majority of patients 3 to 8 weeks after surgery was reversed approximately 2 years after surgery. The majority of patients actually showed a lower atorvastatin systemic exposure long term compared with baseline (Fig. 3; Table 4). Our study emphasizes time after bariatric surgery as an important factor to be considered with regard to systemic drug exposure in these patients.

Few studies have evaluated the effect of current bariatric procedures on systemic drug exposure [7]. To our knowledge, the current study is the first to examine changes in systemic exposure of drugs after biliopancreatic diversion with duodenal switch. Furthermore, the effect of different bariatric procedures on systemic exposure of equivalent drugs was not investigated in the same trial earlier.

In a recent prospective study, Hamad et al. [11] performed repeated measures of drug exposure levels in 12 patients using five different serotonin reuptake inhibitors, both before gastric bypass and three times during the first postoperative year. Most of the patients (8/12) showed a reduced drug AUC 1 month after surgery and normalization to preoperative values after 1 year. This pattern of an initial acute change in exposure levels followed by a return to baseline mirrors the results in the current study.

The opposite direction of the acute change in systemic exposure probably is explained by different properties of the drugs investigated in the respective trials. The antidepressants investigated are mainly metabolized by CYP2D6 and CYP2C19, which represent only a minor portion of the intestinal CYP content [22]. Consequently, these drugs are not metabolized to any relevant degree in the intestinal mucosa. In this situation, a change in absorptive surface area seems to be the most likely explanation for the findings.

On the other hand, atorvastatin is subjected to CYP3A metabolism, which is a major component of the intestinal metabolizing capacity [22]. Hence, bypassing intestinal segments rich in CYP3A enzymes reduces both the intestinal first-pass metabolism and the absorptive surface area. The net result might be an increased systemic exposure due to the outweighing effect of reduced intestinal enzymatic capacity.

Cross-sectional studies of immunosuppressants [25], metformin [19], moxifloxacin [3], sertraline [24], and thyroxine [26] variously report reduced, increased, and unchanged systemic exposure 2 months to 7 years after gastric bypass. The diversity of the findings underscores the importance of properly designed clinical trials. In addition, it indicates that therapeutic effects must be evaluated or serum concentrations measured for each patient postoperatively.

The observed long-term normalization of systemic exposure of atorvastatin acid in the current study may be due to an intestinal adaptation (e.g., by upregulation of CYP3A4) in the small intestine that is more proximally sited after the operations. Investigations after reoperations of obsolete bariatric surgical procedures have shown long-term changes in both enzymatic activity (increased activity of lactase, sucrase, alkaline phosphatase, and thymidine kinase) and morphologic parameters (increased length of intestinal segments and villous hypertrophy) [4, 6, 8, 31, 32].

Adaptation of the intestine also may affect drug bioavailability. Two recent studies have addressed mucosal function and adaptation after gastric bypass. A significant decrease in villous surface area and increased cell proliferation with time was reported in jejunal mucosa just distal to the gastric pouch in eight patients 6 to 8 months after gastric bypass [30]. The authors interpreted these changes as an adaptation of the jejunal mucosa to an appearance more suited for food reception and tissue defense.

Increased cell proliferation in rats that underwent gastric bypass also were shown by le Roux et al. [15]. Additionally, in both rats and humans, they showed dynamic changes in the postprandial rise in glucagon-like peptide-2, a hormone that has a direct effect on bowel mucosa and increases the absorptive surface [5], with a peak at 6 months postoperatively and normalization to preoperative levels at 24 months. This accords with our finding of a long-term dynamic change in systemic exposure of atorvastatin after gastric bypass and duodenal switch.

It has been increasingly evident that CYP3A expression and activity are regulated by nongenetic factors such as endogenous and exogenous chemicals, including steroids and xenobiotics as well as disease states involving cytokines [17, 33]. Accordingly, CYP3A intestinal adaptation may be a possible explanation for the normalization of systemic exposure of atorvastatin acid in the current study.

Our findings may have several other explanations including physiologic changes after bariatric surgery affecting drug bioavailability. The surgery induces a substantial weight loss long term and affects the serum levels of gastrointestinal and systemic hormones [16]. Previous studies also have shown that body weight is associated with intestinal CYP3A activity, and it is conceivable that substantial weight loss after bariatric surgery may induce an increase in CYP3A protein expression over time [9]. In addition, bypassing the small intestine may affect the impact of drug transporters and metabolizing enzymes other than CYP3A on drug bioavailability.

Most of the patients investigated in this study showed changes in systemic exposure of atorvastatin that might be clinically relevant regarding both efficacy and adverse effects. It is conceivable that the results may be extrapolated to other drugs primarily metabolized by CYP3A such as immunosuppressives, calcium-channel blockers, and some antiepileptics, but clinical trials to test each situation are necessary. In addition, the clinical consequences of changes in the systemic exposure of other drugs may differ from those of atorvastatin and therefore warrant close monitoring of patients. For drugs more extensively metabolized by CYP3A, the magnitude of change in systemic exposure might be even greater.

A major limitation of the current study was the relatively small sample size. Nevertheless, to the best of our knowledge, this is the first prospective study to investigate systemic drug exposure in detail more than 1 year after surgery and after two different bariatric surgical procedures. Each patient acted as his or her own control, which directly addressed the changes in systemic exposure after gastric bypass or duodenal switch for the individual across time. Importantly, our results should be considered as hypothesis generating and as requiring verification.

The current study has shown a significant change over time in the systemic exposure of atorvastatin after gastric bypass and duodenal switch, albeit with large inter- and intraindividual variations. Our findings indicate that patients using atorvastatin or drugs with similar pharmacokinetic properties should be monitored closely for both therapeutic and adverse effects, particularly in the case of narrow therapeutic windows. Accordingly, repeated drug dose adjustment may be necessary during the first years after gastric bypass and duodenal switch.
